# Transposon Insertion Finder (TIF): a novel program for detection of *de novo* transpositions of transposable elements

**DOI:** 10.1186/1471-2105-15-71

**Published:** 2014-03-14

**Authors:** Mariko Nakagome, Elena Solovieva, Akira Takahashi, Hiroshi Yasue, Hirohiko Hirochika, Akio Miyao

**Affiliations:** 1 Agrogenomics Research Center, National Institute of Agrobiological Sciences, 2-1-2, Kannondai, Tsukuba, Ibaraki 305-8602, Japan; 2 Current Address: Research Center for Medical Glycoscience, National Institute of Advanced Industrial Science and Technology, Central 2, 1-1-1, Umezono, Tsukuba, Ibaraki 305-8568, Japan

**Keywords:** Transposable elements, Rice, NGS, TSD

## Abstract

**Background:**

Transposition event detection of transposable element (TE) in the genome using short reads from the next-generation sequence (NGS) was difficult, because the nucleotide sequence of TE itself is repetitive, making it difficult to identify locations of its insertions by alignment programs for NGS. We have developed a program with a new algorithm to detect the transpositions from NGS data.

**Results:**

In the process of tool development, we used next-generation sequence (NGS) data of derivative lines (ttm2 and ttm5) of *japonica* rice cv. Nipponbare, regenerated through cell culture. The new program, called a transposon insertion finder (TIF), was applied to detect the *de novo* transpositions of *Tos17* in the regenerated lines. TIF searched 300 million reads of a line within 20 min, identifying 4 and 12 *de novo* transposition in ttm2 and ttm5 lines, respectively. All of the transpositions were confirmed by PCR/electrophoresis and sequencing. Using the program, we also detected new transposon insertions of *P*-element from NGS data of *Drosophila melanogaster.*

**Conclusion:**

TIF operates to find the transposition of any elements provided that target site duplications (TSDs) are generated by their transpositions.

## Background

TEs are mobile genetic elements in the genome. TEs are found in almost all species of prokaryotes and eukaryotes. In eukaryotes, TEs are among the major components of the genome [1]. TE activity is strictly controlled, and almost all of the TEs in the genome are inactive. Under stress or other special conditions, TEs may be activated to transpose into other locations within the genome [2,3]. TEs are categorized into two classes, class I (RNA type) transposons, retrotransposons, and class II (DNA type) transposons. Class I transposons are transposed using the ‘copy-and-paste’ manner through reverse transcription of their transcripts, whereas the class II transposons are transposed in the ‘cut-and-paste’ manner. Class I transposons are categorized into two sub types in terms of the presence or absence of long terminal repeat (LTR). On the other hand, class II transposons have terminal inverted repeat (TIR). The transposition of class I transposons containing LTR and class II DNA transposons create less than 10 base pair (bp) target site duplications (TSDs) [4], whereas that of the class I transposon containing no LTR does not.

*De novo* transposition of TEs plays an essential role in genome-wide structural change, leading to phenotypic changes. More than 30 programs have been reported to be available for detection of TE loci in genomes [5]. However, it was difficult to detect the new insertion events of TEs using the programs reported previously. For more efficient detection of *de novo* transpositions, several programs have been developed; those are Next-generation VariationHunter, BreakDancer, ngs_te_mapper, RelocaTE, RetroSeq, PoPolation TE, and TE-locate [4,6-11]. However, since these programs have often given not-consistent results [12], a new program giving more convincing results must be developed.

*Tos17* is a class I Ty1-*copia*-type retrotransposon, 4.1 kb in length, and generates 5 bp TSDs in its insertion events in *Oryza sativa*. Since *Tos17* transposition occurs in a ‘copy-and-paste’ manner, the copy number of *Tos17* is increased by transposition. *Tos17s* are actively transposed in the genomes of cultured cells, and the transposed *Tos17s* are inactivated in plants regenerated from cultured cells [13]. On average, approximately 10 new copies of *Tos17* are transposed during 5 months of cell culture in *japonica* rice cv. Nipponbare [13,14]. Since *Tos17* transposition may cause gene disruption in *Oryza sativa*[15], fifty thousand lines containing possible new *Tos17* insertions have been created, and their phenotypic traits have been evaluated in the field [16]. Thus, identification of the transposed position is essential for determination of genes responsible for the traits. Although the TAIL-PCR and suppression PCR, followed by dye-deoxy terminator sequencing, have been used for the detection of the *Tos17* transposed site, they contain intrinsic detection limitations, due to by chance amplification using the randomly-chosen primer for TAIL-PCR or the recognition sites of restriction enzymes for suppression PCR [14,17].

An additional way to detect the transposed position would be direct analysis of whole genome sequence data. However, since the detection programs have not yielded consistent results as described above, a new program TIF has been developed in the present study for detection of *Tos17 de novo* transpositions in established rice lines. We applied TIF for the *de novo* transpositions in 2 rice lines using NGS data and validated the results of TIF analysis by PCR/electrophoresis and sequencing of PCR products in comparison with RelocaTE, which has been shown to be suitable for the present analysis of *Tos17-*transposed regenerated plants. In addition, we demonstrated that TIF is applicable for detection of *de novo P*-element insertions using NGS data of *D. melanogaster.*

## Methods

### TIF algorithm

**Figure 1:**
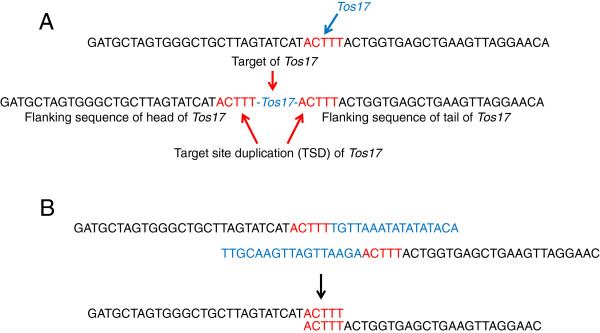
**Schematic presentation of insertion of *Tos17 *and TSD and principal of TIF algorithm. A**. In the process of *Tos17* insertion*,* 5 bp sequence (shown in red letters) flanking *Tos17* is duplicated. **B**. Short reads of NGS containing end of *Tos17* sequence (shown in blue letters) were searched and then made a group by TSD.

In the event of a *Tos17* insertion as illustrated in Figure 1A, the 5 base pair at the cleavage point is duplicated. We designed two algorithms; both were designed to select reads containing ends of TE by focusing on TSDs (Figure 1B).

Algorithm 1

(1)  Sequences in FASTQ format data containing 5′-end (head) or 3′-end (tail) sequences of the target TE are selected using a search tool with regular expression.

(2)  The sequences flanking the junction of the TE are extracted and grouped by TSDs. The longest pair of flanking sequences in each TSD group is selected.

(3)  The locations of the sequences flanking the head and tail sequence of the TE are identified by BLAST search against the reference genome sequence.

Algorithm 2

(1)  Sequences in FASTQ format data containing 5′-end (head) or 3′-end (tail) sequences of the target TE are selected using a search tool with regular expression. (Same as the algorithm 1)

(2)  The locations of the sequences flanking the head and tail sequence of the TE are identified by BLAST search against the reference genome sequence.

(3)  Read pairs, the distance of which between their loci of TE junctions on the reference genome is less than 10 bp, are then selected, and subjected to examination of whether the read pairs contain TSD. When read pairs are found to contain TSD, they are scored as candidates having TEs.

**Figure 2:**
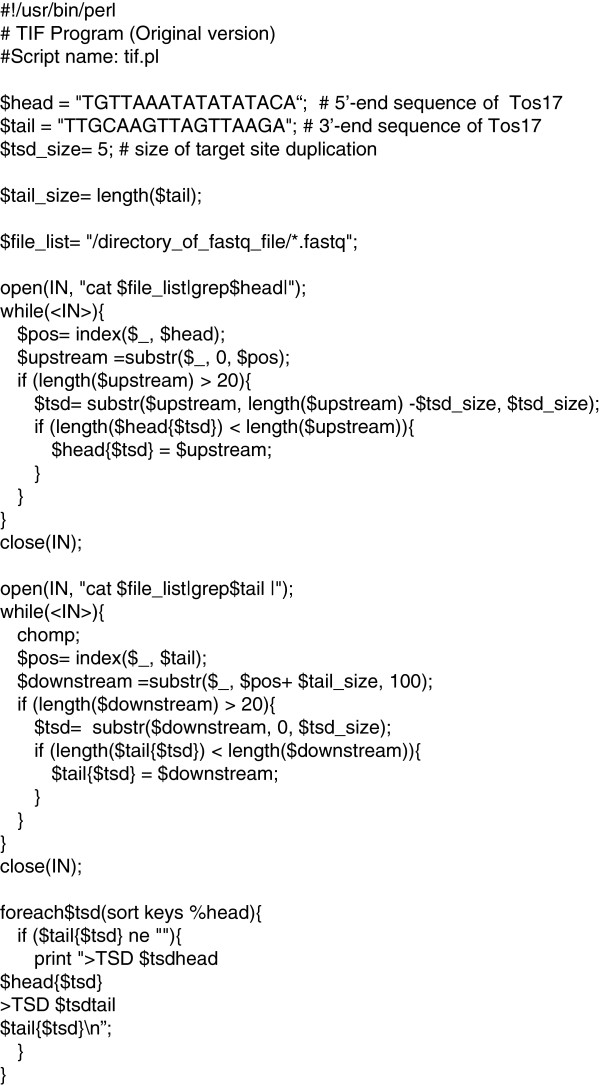
**Basic TIF algorithm.** Input sequences are short read sequences of a sequencer such as Illumina HiSeq2000 with FASTQ format. The data are outputted with FASTA format.

In the first step, both algorithms select reads containing junctions of TE. Since algorithm 1 selects read pairs containing TSD in the second step, if the TSD length is unknown, algorithm 2 should be used. In summary, algorithm 1 requires the information of TSD length but not the reference genome sequence to detect target sequence of TE insertion event. Algorithm 2 does not require the information of TSD length, but rather the reference genome sequence. The basic version of TIF (Basic TIF) was based on algorithm 1 as is shown in Figure 2; and the extended version (extended TIF), based on algorithm 2 (not shown in the figure). Both programs can be obtained from https://github.com/akiomiyao/tif.

### NGS data for TIF and RelocaTE analysis

NGS data of Nipponbare and its derivative lines (NC7756-1, an individual of ttm2 mutant, and ND6834-21, an individual of ttm5 mutant: the Rice Genome Resource Center (RGRC) at http://www.rgrc.dna.affrc.go.jp) regenerated through cell culture were obtained from GenBank (Short Read Archive SRP000719, SRX183508, SRR556173, SRX183509, SRR556174, SRR556175, at http://www.ncbi.nlm.nih.gov/sra). Briefly, the NGS data were generated using Illumina Hiseq 2000 sequencing system [18]. The reads in the NGS data are 100 bp length paired-end reads. In this report, ttm2 and ttm5 were used for abbreviations of NC7756-1 and ND6834-21, respectively. The numbers of reads for ttm2 and ttm5 were 292,333,698 and 294,687,288, respectively. The IRGSP1.0 reference rice genome sequence was obtained from http://rapdb.dna.affrc.go.jp/download/irgsp1.html[17,19]. RelocaTE was obtained from https://github.com/srobb1/RelocaTE. For the analysis of *P*-element transposition in *D. melanogaster,* SRR823377 and SRR823382 were obtained from the Short Read Archives [20]. The reference genome of *D. melanogaster* was obtained from ftp://ftp.flybase.net/, and the file of dmel-all-chromosome-r5.55.fasta was used as the reference genome sequence. BLAST programs used blastall 2.2.26 and Nucleotide-Nucleotide BLAST 2.2.29+. Parameters for blastall command were ‘-p blastn -d IRGSP1.0 -m 8 -b 1’; and those for blastn (BLAST 2.2.29+), ‘-db IRGSP1.0 -outfmt 6 -num_alignments 1’.

### Validation of TIF and RelocaTE output data using PCR/electrophoresis and DNA sequencing

In order to design primer pairs for amplification of transposed *Tos17* together with its flanking sequences, the output in multiple-FASTA format was subjected to BLASTN against the reference genome of rice (IRGSP1.0), and primer pairs were designed in the reference sequence across the target site of *Tos17* using the Primer3 (release 1.1.4) program with default settings [21]. The primer pairs thus obtained were used together with *Tos17*-tail3 (GAGAGCATCATCGGTTACATCTTCTC) or *Tos17*-tail5 (CATCGGA-TGTCCAGTCCATTG) primer to perform “triple-primer” PCR (http://signal.salk.edu/tdnaprimers.2.html) using ttm2 and ttm5 genomic DNAs, which were purified from leafs of NC7756-1, and ND6834-21 by cetyl trimethyl ammonium bromide (CTAB) method [22]. Target sites of *Tos17* insertions are amplified using GoTaq Green Master Mix (Promega) and GeneAmp PCR System 9700 (Applied Biosystems) with 30 cycles of 94°C 15 sec, 60°C 30 sec, and 72°C 2 min.

The amplified fragments were electrophoresed in 1.5% agarose gels to identify the fragments possibly containing *Tos17* sequence based on their sizes. The fragments thus identified were then extracted using the Wizard SV Gel and PCR Clean-Up System (Promega), and were sequenced using a BigDye terminator v3.1 Cycle Sequencing Kit and a 3130xl Genetic Analyzer (Applied Biosystems).

## Results

### Performance of TIF and RelocaTE

The performance tests of basic TIF and RelocaTE were done using the ttm2/ttm5 reads, and a computer equipped with the Intel Xeon Processor E5620@2.4 GHz, 32 GB memory under the CentOS 6.2 operating system. The input file “mping.fa” of RelocaTE was substituted with the file “tos17.fa” for detection of *Tos17* insertions in rice genomes. The output of the time command after analysis of the reads was shown in Table 1, revealing that TIF performance was more than 5 times higher than RelocaTE.

**Table 1:** Run time (Real) for basic TIF and RelocaTE

	**Number of reads**	**TIF**	**RelocaTE**
ttm2	292 333 698	20 m 3.811 s	103 m 40.315 s
ttm5	294 687 288	18 m 0.557 s	105 m 16.471 s

### TIF output

**Figure 3:**
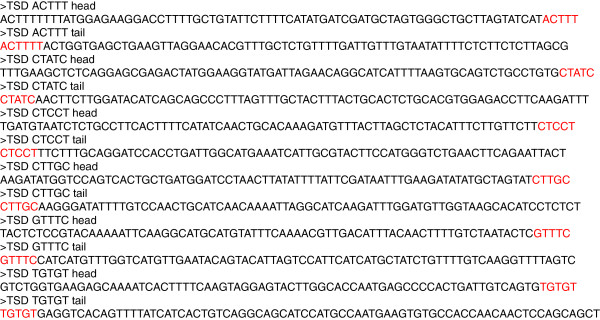
**TIF output of short reads for ttm2.** FASTQ files of ttm2 are directly subjected to TIF program. TSDs were shown in red letters.

The data are outputted in multiple-FASTA format sorted by TSD sequence. The output can be subjected directly to BLAST search [23]. The basic TIF output of ttm2 is shown in Figure 3 as an example. All TIF outputs, those of ttm2 and ttm5, and their BLASTN search results are shown in Additional file 1, and all RelocaTE outputs, in Additional file 2.

### Optimization of length of head, and tail sequence, and length of TSD

Since a high resolution reference sequence of rice was available, sensitivity and specificity of TIF algorithms were examined first using extended TIF (algorithm 2), together with BLAST 2.2.26 and BLAST 2.2.29+. The examination results were shown in Table 2.

**Table 2:** Sensitivity and specificity of TIF

		**ttm2**			**ttm5**		
		^**a**^**FASTA**	^**b**^**Loci**		**FASTA**	**Loci**	
**Head and tail (bp)**	**TSD (bp)**		^**c**^**2.2.26**	^**d**^**2.2.29+**		**2.2.26**	**2.2.29+**
21	5	28	4	3	52	11	10
20	5	28	4	3	56	12	11
19	5	29	4	3	56	12	11
18	5	29	4	3	57	12	11
17	5	34	4	3	63	12	11
16	5	43	4	3	74	12	11
15	5	93	3	2	122	12	11
14	5	167	3	2	190	12	11
13	5	424	3	2	433	12	11
12	5	1616	3	2	1677	12	11
11	5	4038	3	2	4142	12	11

^a^Numbers of flanking sequences detected with head or tail sequences. ^b^Numbers of detected loci on the reference genome by ^c^BLAST 2.2.26 and ^d^BLAST 2.2.29 + .

When the length of head and tail sequences was 17 or less, the number of flanking sequences containing head or tail sequence increased. The increase was considered to be the result of the reduction of sequence specificity in head or/and tail sequences. At a length of 21, the number of detected loci was decreased in ttm5. The decrease was considered to be the result of a spontaneous mutation in the tail sequence, which was found by comparison between the reference and the selected read. BLAST 2.2.29+ returns smaller number of loci than BLAST 2.2.26. The loci demonstrated by BLAST 2.2.26 and BLAST 2.2.29+ were examined by PCR and sequencing, demonstrating that BLAST 2.2.26 gives more accurate results than BLAST 2.2.29+ in our present study (see below). Concerning the TSD, all TSDs detected with algorithm 2 were found to be 5 bp in length.

The basic TIF (algorithm 1) requires the TSD length instead of the reference genome sequence. The result of basic TIF with various lengths of TSD is shown in Table 3. The basic TIF analysis of ttm2 with 5 bp TSD length gave the target loci number consistent with that obtained from the extended TIF analysis. As for ttm5, the basic TIF analysis gave 11 loci, which are one locus smaller than those (12 loci) obtained from the extended TIF analysis. This was the result of the TSD sequence coincidence between original *Tos17* and one of transposed *Tos17* which was confirmed by PCR and sequencing as described below.

**Table 3:** Specificity of TSD length by basic TIF

		**ttm2**		**ttm5**	
**Head and tail (bp)**	**TSD (bp)**	**FASTA**	**Loci**	**FASTA**	**Loci**
17	3	4	0	12	2
17	4	2	0	2	0
17	5	12	4	24	11
17	6	0	0	0	0
17	7	0	0	0	0

### *De novo* insertion site of *Tos17* in the genome

The extended TIF with BLASTN (2.2.26) search against the reference genome sequence of Nipponbare (IRGSP1.0) was used for detection of *de novo* insertion of *Tos17* in ttm2 and ttm5. The flanking TSDs to *Tos17* and their insertion sites detected by the extended TIF and RelocaTE were assigned to the Nipponbare genome sequence (Table 4). All lengths of TSD detected by the extended TIF and RelocaTE were 5 bp. For ttm2, the results of TIF were exactly the same as those of RelocaTE. For ttm5, 12 *Tos17* TSDs were detected with TIF; and 8, with RelocaTE: RelocaTE failed to detect 4 *Tos17* TSDs out of 12 detected by TIF, and TIF failed to detect one *Tos17* TSD out of 8 detected by RelocaTE.

**Table 4:** **Assignment of TSDs flanking transposed **^**a**^ ***Tos17 *** **in ttm2 and ttm5 to the genome of the original line, Nipponbare**

**Detected by**		**Line**	**Chromosome**	^**b**^**Position of junction for**		**Size of TSD**	**TSD for**		**Direction**	**Confirmed by PCR/Sequencing**
				***Tos17 *** **tail**	***Tos17 *** **head**		***Tos17 *** **tail**	***Tos17 *** **head**		
TIF	RelocaTE	ttm2	chr04	30 259 052	30 259 056	5	GTTTC	GTTTC	Forward	Yes
TIF	RelocaTE	ttm2	chr05	1 925 905	1 925 909	5	CTATC	CTATC	Forward	Yes
TIF	RelocaTE	ttm2	chr10	22 134 718	22 134 714	5	CTTGC	CTTGC	Reverse	Yes
TIF	RelocaTE	ttm2	chr10	22 531 003	22 531 007	5	ACTTT	ACTTT	Forward	Yes
TIF		ttm5	chr01	34 453 645	34 453 641	5	CTTTG	CTTTG	Reverse	Yes
TIF	RelocaTE	ttm5	chr02	1 004 769	1 004 765	5	ATACC	ATACC	Reverse	Yes
TIF	RelocaTE	ttm5	chr02	31 596 628	31 596 632	5	CTAAT	CTAAT	Forward	Yes
TIF	RelocaTE	ttm5	chr03	741 226	741 222	5	GCTGC	GCTGC	Reverse	Yes
TIF	RelocaTE	ttm5	chr03	8 304 678	8 304 674	5	GAATA	GAATA	Reverse	Yes
TIF		ttm5	chr06	24 967 881	24 967 877	5	TGCAT	TGCAT	Reverse	Yes
TIF		ttm5	chr07	20 064 391	20 064 395	5	CTTAT	CTTAT	Forward	^c^Yes
				20 080 552	20 080 556					
TIF	RelocaTE	ttm5	chr09	12 970 618	12 970 614	5	CATGC	CATGC	Reverse	Yes
	RelocaTE	ttm5	chr10	14 739 090	14 739 094	5		GAACT	Forward	No
TIF		ttm5	chr10	19 069 885	19 069 889	5	ACTTG	ACTTG	Forward	Yes
TIF	RelocaTE	ttm5	chr10	21 583 054	21 583 058	5	CTTAT	CTTAT	Forward	Yes
TIF	RelocaTE	ttm5	chr12	2 155 899	2 155 895	5	GGAAC	GGAAC	Reverse	Yes

^a^Original *Tos17s* are located from 26 694 799 to 26 698 904 at chromosome 7 and from 15 415 378 to 15 419 573 at chromosome 10.

^b^positions of 1^st^ base of flanking sequences to the tail and head sequence of *Tos17.*

^c^TSD on chromosome 7 in ttm5 is located within one of two very similar sequences; thus, it was difficult to determine its position conclusively from the short read sequences.

### Validation of TIF and RelocaTE outputs

**Figure 4:**
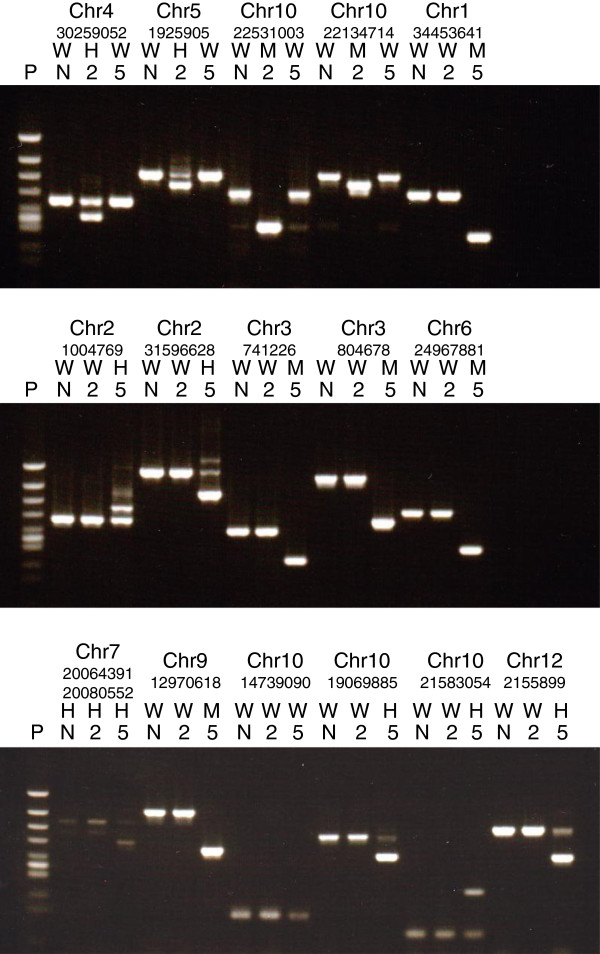
**Confirmation of *Tos17 *insertions detected by TIF and RelocaTE programs with PCR/electrophoresis.** Genomic DNAs of ttm2 and ttm5 were subjected to PCR using the triple-primer method (see Methods), and the PCR products were electrophoresed in 1.5% agarose gels with molecular weight markers, followed by detection of amplified fragments with ethidium bromide. P represents molecular weight marker of φX174/*Hin*cII (Toyobo, Osaka, Japan); N, Nipponbare; 2, ttm2; 5, ttm5; W, wild-type; M, homozygous *Tos17* insertion; H, heterozygous *Tos17* insertion. *Tos17* transposed loci are indicated by chromosome number and the start position of the TSDs on genome sequence.

All locations corresponding to line-specific insertion events were subjected to amplification of DNA fragments from ttm2 and ttm5 genomic DNAs using the triple-primer PCR method described in Methods, and the amplified fragments were electrophoresed to determine their sizes. As electropherograms were shown in Figure 4, the *Tos17* insertions indicated by fragment size-shifts were found to be compatible with those revealed by the TIF output (Table 4). However, in the *Tos17* insertion site on chromosome 10 indicated by RelocaTE but not by TIF, no size-shift was observed in the amplified fragments, indicating that no *Tos17* insertion occurred in the site. All fragments showing size-shifts were purified and sequenced with a capillary sequencer. The fragments thus sequenced were found to contain *Tos17* junction sequences (Additional file 3: Figure S1). These results may be summarized as follows: 1) 4 *Tos17* insertion sites in ttm2 revealed by TIF and also by RelocaTE were shown to contain *Tos17* sequences; 2) 7 *Tos17* insertion sites in ttm5 revealed by TIF and also by RelocaTE, contained *Tos17* sequences; 3) the remaining 4 *Tos17* insertion sites in ttm5 revealed by TIF, contained *Tos17* sequences; and 4) one *Tos17* insertion site in ttm5 revealed by RelocaTE, contained no *Tos17* sequence. In conclusion, TIF is able to effectively detect *Tos17* insertion sites in rice lines with higher specificity and sensitivity than RelocaTE.

### Application TIFs to detect *P*-element insertion events in NGS of *D. melanogaster*

Since the reference genome sequence of *D. melanogaster* was available, we first applied the extended TIF for identification *P-*element insertions in the genomes, NGS data of SRR823377 and SRR823382. The parameters for the analysis were as follows: *P-*element head sequence was CATGATGAAATAACATA; and tail sequence, TATGTTATTTCATCATG. The number of *P-*element insertions was found to be 137 for SRR823377, and 222 for SRR823382 (shown in Additional file 4): Eighty-eight common insertion sites were found in SRR82377 and SRR823382. Forty-nine and 134 sites were found specifically in SRR82377 and SRR823382, respectively. All lengths of TSD detected by extended TIF were 8 bp. Then, we performed basic TIF analysis for SRR82377 and SRR823382 using the head and tail sequences and 8 bp as TSD parameter; revealing 85 common insertion sites in SRR82377 and SRR823382, 53 sites specifically in SRR82377, and 131 sites in SRR823382.

## Discussion and conclusion

A program designated as “TIF” was developed to detect *de novo* TSD-based transposition of TEs using NGS data. The premise is quite simple: reads containing 5′- or 3′-end of the TE are selected and grouped by TSD sequence. We developed two types of TIF, basic and extended TIFs, algorithms of which were based on the same concept. The both algorithms are to select reads containing 5′-end of or 3′-end of the TE in the first step. The second step of our algorithm involves pairing of selected reads for corresponding insertion of TE. The basic TIF selects reads based on their TSD sequence for the pairing, which are applicable for NGS of genomes without their reference genome sequences, provided that length of TSD and 17 bp terminal sequence of TE are known. Alternatively, the extended TIF selects reads based on their assigned locations on the reference genome, which are applicable for NGS of genomes without TSD information, provided that their reference genome sequences and 17 bp terminal sequence of TE are known.

The programs reported earlier, ngs_te_mapper, RelocaTE, RetroSeq, PoPloolationTE, and TE-locate, require the mapped locations of reads on the reference genome sequence in the initial step of analysis. It is, therefore, impossible to obtain information about TE insertion in the repetitive regions. However, since the basic TIF does not require the locations of reads in the reference genome, TE insertions even in the repetitive regions are able to be identified. The insertions in repetitive regions may not function in phenotypic change, but may be used as genetic markers.

RelocaTE and ngs_te_mapper resembled the basic TIF in terms of the usage of TSD information, so that we attempted to compare the basic TIF with those programs. Currently, since Relocate was available on the website, we compared the performance of the basic TIF with that of RelocaTE. The comparison demonstrated that, as described in the Results section, TIFs are able to detect *Tos17* insertions more accurately than RelocaTE. In addition, the performance time of TIF is shown to be 5 times shorter than that of RelocaTE in a given job.

In the case of *Tos17,* as the length of the TSD is 5 bp, it is possible to calculate the probability that the flanking sequences of differently located *Tos17s* are assigned to one and the same *Tos17* locus as being 1/1024. However, since approximately 10 *Tos17* transposition events were shown to occur in the lines examined [14], the possibility of mis-assigning reads to the wrong insertion site by the basic TIF is considered to be low.

In the analysis of TEs with shorter TSDs and/or a high frequency of transposition, however, the mis-assignment would not be negligible. For such analysis, extended TIF based on the algorithm 2 is developed. The extended TIF examines all combinations of flanking sequences detected by BLAST search against the reference sequence; leading to no mis-assignment that possibly occurs in the basic TIF. However, it is basically impossible to identify the insertions with the extended TIF in the case that the TEs are located in the sequence showing completely the same with that in other locations, due to the BLAST search integrated in the extended TIF. When run times of the basic and extended TIFs were compared using the NGS of ttm2, the run times of the two programs showed little difference, if any (data now shown).

To evaluate performance of TIF algorithms for other spices, we investigated the *P-*element insertions in two NGS data of *D. melanogaster* (SRR823377 and SRR823382)*.* The basic as well as extended TIFs detected almost the same insertions as described in the Results, indicating that the TIFs are applicable to detect transposition events in various species using NGS data. In addition, information of *P-*element insertions among recombinant inbred lines will help mapping of phenotypic trait and/or the isolation of insertion mutant for target phenotype.

In conclusion, basic/extended TIFs is a powerful tool to detect *de novo* transposed sites of TEs using NGS data.

## Availability of supporting data

Scripts of basic and extended TIF and demonstration data can be obtained from https://github.com/akiomiyao/tif.

## Abbreviations

TIF: Transposon insertion finder; TSD: Target site duplication; LTR: Long terminal repeat; TIR: Terminal inverted repeat; NGS: Next-generation sequence.

## Competing interests

The authors declare that they have no competing interests.

## Authors’ contributions

AT selected and provided mutant line. AM developed TIF algorithm and program, carried out the informatics studies, and drafted the manuscript. ES assisted informatics study. MN carried out the experiment for confirmation of TIF and RelocaTE output results. HH and HY contributed to the discussion and preparation of the manuscript. All authors read and approved the final manuscript.

## Supplementary Material

Additional file 1 **Result of basic TIF (shown in Figure **2**) and BLAST search.** Click here for file

Additional file 2 Outputs of RelocaTE. Click here for file

Additional file 3: Figure S1 Chromatograms of capillary sequencer around the junction. Click here for file

Additional file 4 **Detection of P-element insertion in *D. melanogaster.***
Click here for file
