# Supplementary material for: Transposon Insertion Finder (TIF): a novel program for detection of de novo transpositions of transposable elements
Source: BMC Bioinformatics. 2014 Mar 14;15:71. doi: 10.1186/1471-2105-15-71 (PMC4004357; doi:10.1186/1471-2105-15-71)
Supplement: Additional file 3: Figure S1 — Chromatograms of capillary sequencer around the junction. [file 1471-2105-15-71-S3.pptx]

## Slide 1
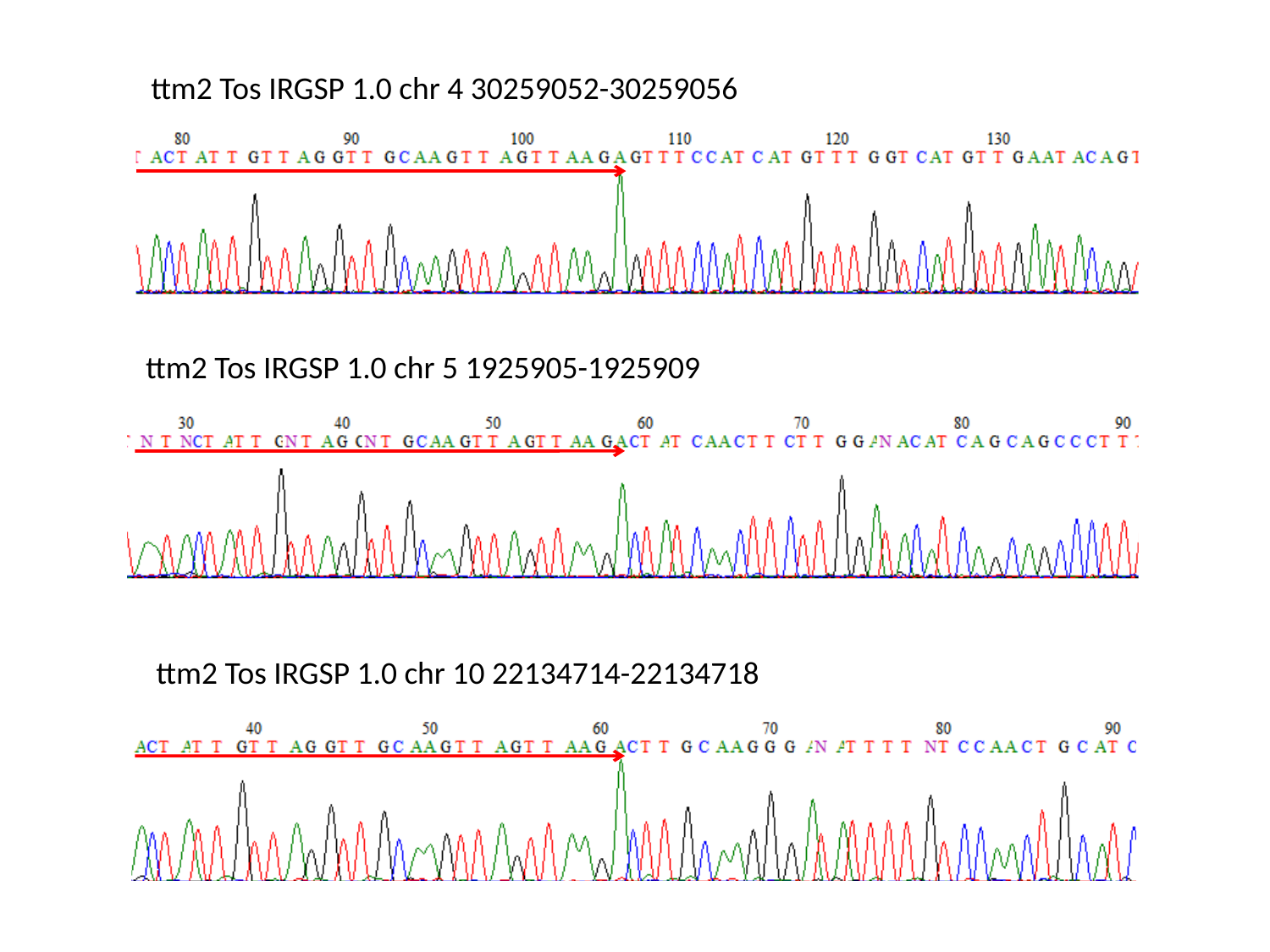

ttm2 Tos IRGSP 1.0 chr 4 30259052-30259056
ttm2 Tos IRGSP 1.0 chr 5 1925905-1925909
ttm2 Tos IRGSP 1.0 chr 10 22134714-22134718

## Slide 2
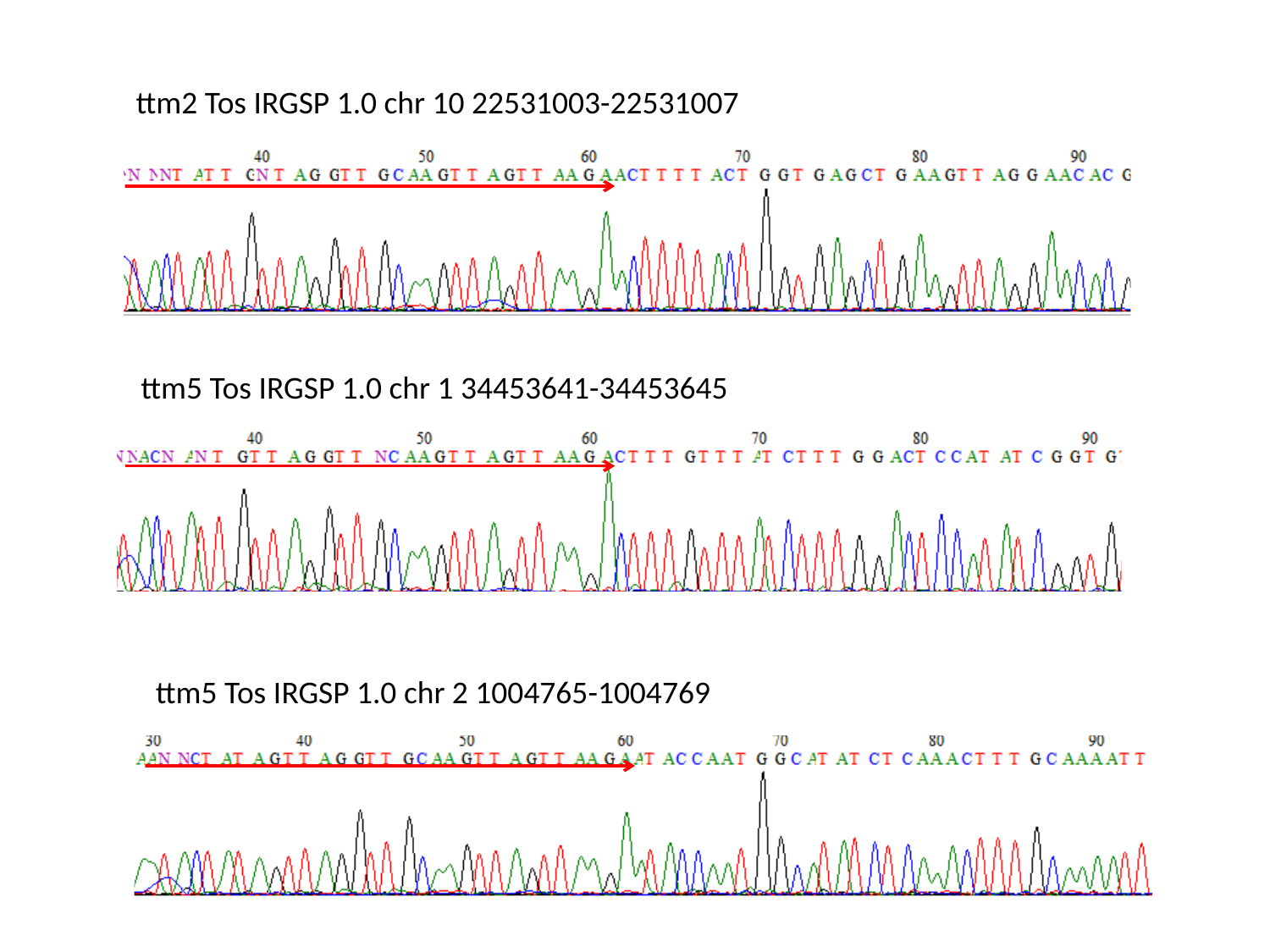

ttm2 Tos IRGSP 1.0 chr 10 22531003-22531007
ttm5 Tos IRGSP 1.0 chr 1 34453641-34453645
ttm5 Tos IRGSP 1.0 chr 2 1004765-1004769

## Slide 3
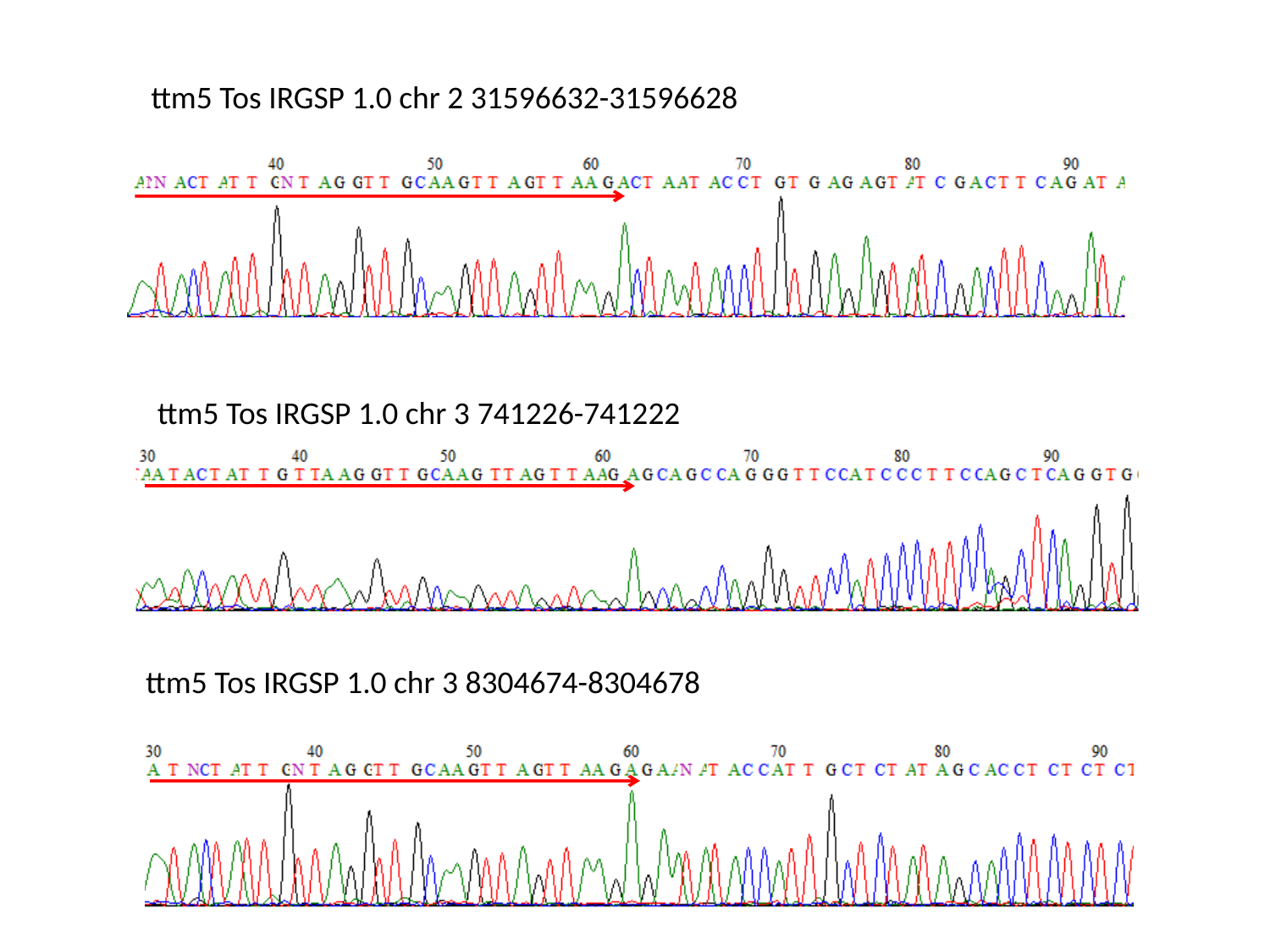

ttm5 Tos IRGSP 1.0 chr 2 31596632-31596628
ttm5 Tos IRGSP 1.0 chr 3 741226-741222
ttm5 Tos IRGSP 1.0 chr 3 8304674-8304678

## Slide 4
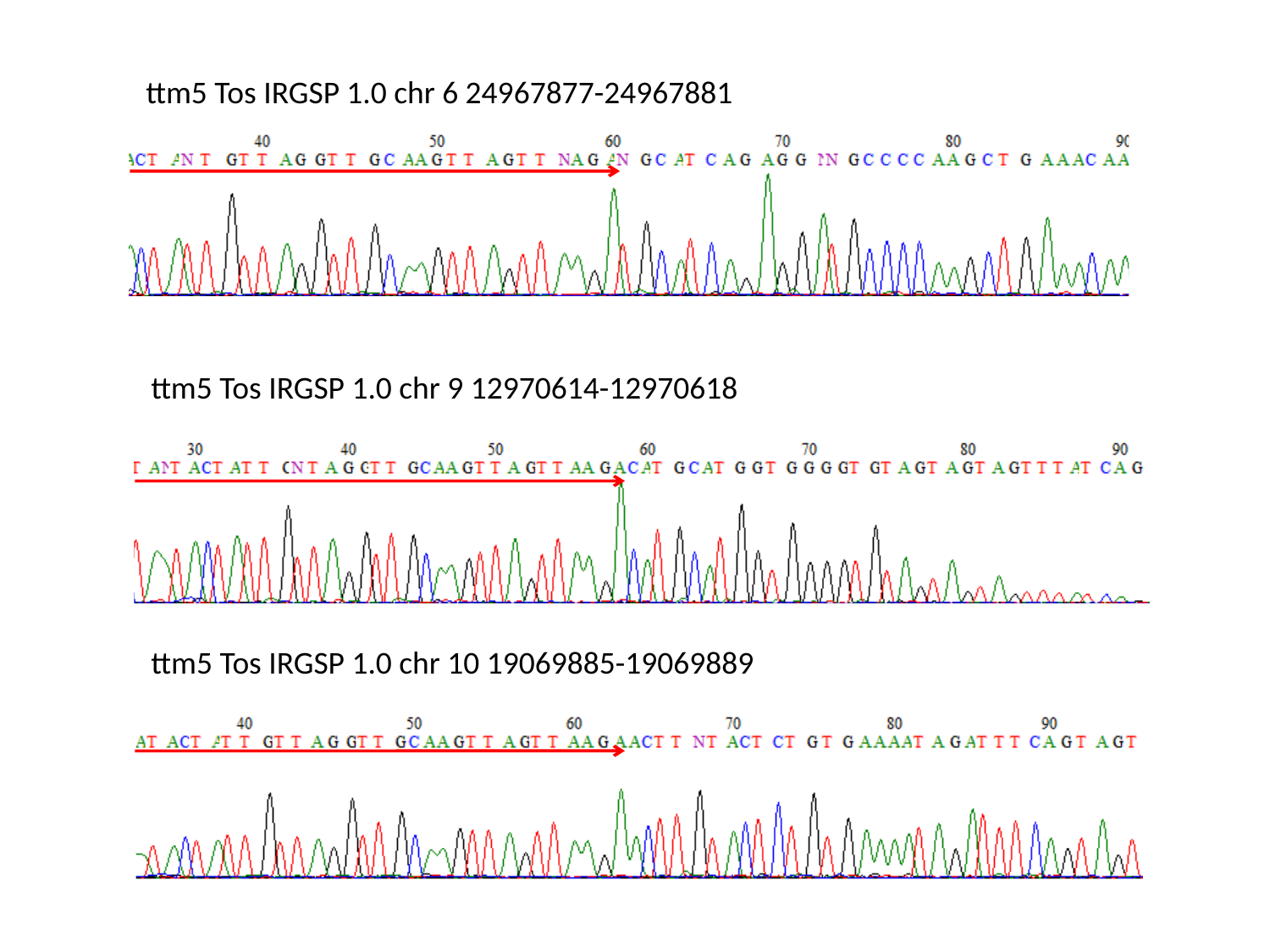

ttm5 Tos IRGSP 1.0 chr 6 24967877-24967881
ttm5 Tos IRGSP 1.0 chr 9 12970614-12970618
ttm5 Tos IRGSP 1.0 chr 10 19069885-19069889

## Slide 5
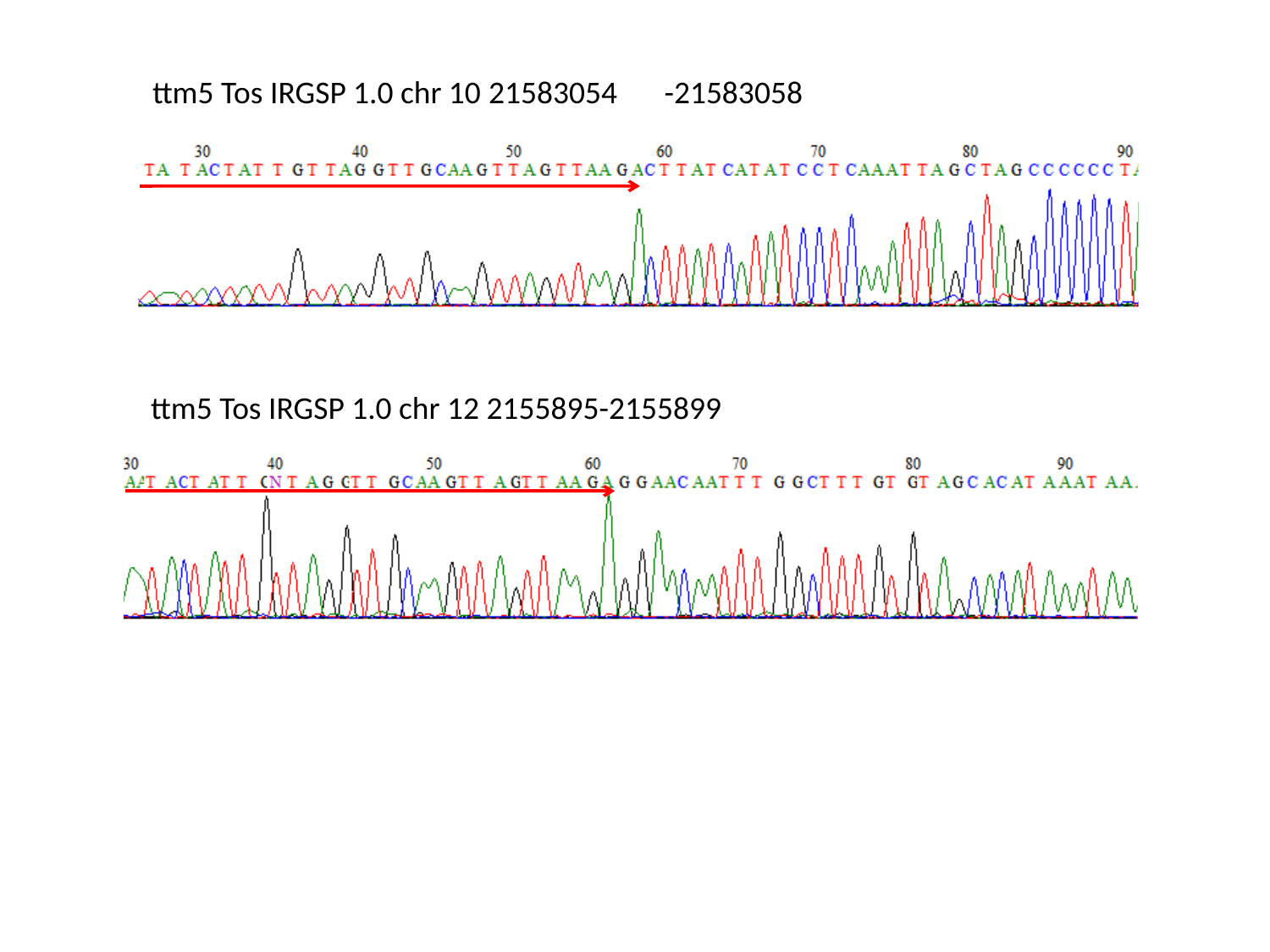

ttm5 Tos IRGSP 1.0 chr 10 21583054　-21583058
ttm5 Tos IRGSP 1.0 chr 12 2155895-2155899
